# *Do not play God*: contrasting effects of deontological guilt and pride on decision-making

**DOI:** 10.3389/fpsyg.2015.01251

**Published:** 2015-08-25

**Authors:** Alessandra Mancini, Francesco Mancini

**Affiliations:** ^1^Scuola di Specializzazione in Psicoterapia Cognitiva S.r.l. – Associazione di Psicoterapia CognitivaRome, Italy; ^2^Guglielmo Marconi UniversityRome, Italy

**Keywords:** guilt, altruistic guilt, deontological guilt, pride, ultimatum game, “Do not play God” principle

## Abstract

Recent accounts support the existence of two distinct feelings of guilt: altruistic guilt (AG), arising from the appraisal of not having been altruistic toward a victim and deontological guilt (DG), emerging from the appraisal of having violated an intuitive moral rule. Neuroimaging data has shown that the two guilt feelings trigger different neural networks, with DG selectively activating the insula, a brain area involved in the processing of disgust and self-reproach. Thus, insula activation could reflect the major involvement of self-reproach in DG rather than in AG. However, only a few studies have empirically tested whether and how DG and AG differently affect decision making and none have compared enhanced self-worth. Here we asked three groups of participants, respectively, induced with either pride, DG or AG, to participate in a third-party version of the ultimatum game in which they were asked to decide on behalf of others to accept or reject economic offers with several degrees of fairness. Results revealed that only deontological participants had higher median acceptances of Moderately Unfair offers as compared to proud participants. However fairness judgments were not different between groups, suggesting that deontological participants’ moral standards had not decreased. Crucially, a higher increase in DG was associated with an increase in the odds of accepting 30:70 offers. The opposite effects that DG and pride exert on self-worth can account for these results. Specifically, proud participants felt entitled enough to take action in order to restore equity, while deontological participants followed the “Do not play God” principle, which limited their decisional autonomy, not allowing them to decide on behalf of others.

## Introduction

“Impious myself, and from an impious race.

Where is my splendor now?”

(Oedipus the King, Sophocles)

Traditional approaches to guilt tend to describe a unique emotion with specific situational and psychological determinants, a precise behavioral drive and a clear evolutionary function. However, different models lead to different explanations of the same emotional phenomenon, namely guilt. According to the *intrapsychic or psychoanalytic* approach (Mosher, 1965, 1966; Lewis, 1971; Piers and Singer, 1971; Wertheim and Schwarz, 1983; Monteith, 1993), guilt is the emotional result of a conflict between our interiorized moral authority and our behaviors (Fromm, 1985). Its evolutionary function is to keep human behavior in line with moral standards (Freud, 1959, 1961a,b). In this view, guilt corresponds to the feeling of having disobeyed one’s own inner moral values. This might cause an expectation or fear of punishment (Wicker et al., 1983), the tendency to atone or the will to apologize (Nelissen and Zeelenberg, 2009). The person who feels guilty has the feeling of being a “bad person” (Lewis, 1971).

Conversely, following the *interpersonal theory* (Hoffman, 1981, 1987; Baumeister et al., 1994; Niedenthal et al., 1994; Tangney, 1999; Tangney and Dearing, 2002; Ketelaar and Au, 2003), guilt results from the awareness of having caused unjustified harm to another or, in a more general sense, not having behaved altruistically. This feeling is based on empathy and compassion (Hoffman, 1981; Baumeister et al., 1994; Niedenthal et al., 1994; O’Connor et al., 1999, 2000; Tangney, 1998). Here, the trigger is a suffering person, unjustly penalized, who we did not help or did not even try to share his or her pain. The evolutionary function of altruistic guilt (AG) is to establish non-aggressive relationships and its aim is to protect and maintain reciprocal altruism and to restore equity (Trivers, 1971; Baumeister et al., 1994).

Even if both approaches describe guilt as a unique feeling, it is clear that they show important contrasting elements both on the level of motivation and on action tendencies. In particular, the psychoanalytic approach focuses on the fear of punishment (Tomkins, 1963; Mosher, 1965; Lewis, 1971), which should induce avoidant behavior. In contrast, according to the interpersonal approach, guilt should promote altruistic tendencies as well as a closeness to the putative “victim.” The question therefore is the following: is guilt a single emotion that activates contrasting behavioral outcomes depending on different situational variables, or are we to consider the existence of two distinct senses of guilt? (Carnì et al., 2013).

A recent attempt has been made (Mancini, 2008) to integrate these two perspective in a comprehensive model. According to this approach the intrapsychic and interpersonal models are not mutually exclusive if we consider the existence of two independent emotions, namely deontological guilt (DG) and AG. The word *independent* here signals that although these two kinds of guilt can be (and often *are*) experienced at the same time, they can be conceptually distinguishable with reference to appraisal theories of emotion, according to which specific cognitions are important antecedents of specific emotions and thereby of specific action tendencies (Smith and Ellsworth, 1985; Oatley and Johnson-Laird, 1987, 1992; Frijda et al., 1989; Scherer, 1999, 2001). In particular, in AG there is always a victim that suffers harm and the appraisal of not having been altruistic, but there may not have been any violation of moral rules (Baumeister et al., 1994). In DG, on the contrary, there might not have been a victim at all and the appraisal of having violated an intuitive moral rule is necessary and sufficient (Mancini, 2008; Gangemi and Mancini, 2011). Therefore, the two feelings are not just triggered by different types of events, but differ only in virtue of the appraisal of the event in the context of individual goals (Carnì et al., 2013). In particular, DG emerges from the appraisal of having disobeyed moral authority, or a deontological norm such as “Do not play God.” According to this norm, no one has the right to make a decision that does not respect the limits of his/her social rank or function (Sunstein, 2005). Therefore, the model predicts feelings of unworthiness and the expectation, or fear, of a punishment (Mancini, 2008; Gangemi and Mancini, 2011). Punishment is an essential ingredient for this type of guilt because, through the punishment of the transgressor, rank differences between him/her and authority are restored (Mancini, 2008). AG appears when one appraises his/her own conduct as not altruistic. The emotional state is connected to empathy, sorrow, and compassion for the victim. According to the model, AG should imply behavioral tendencies aimed at minimizing the number of victims, alleviating their suffering and restoring equity (Baumeister et al., 1995; Ketelaar and Au, 2003; Kubany and Watson, 2003). Indeed its evolutionary function is to maintain, reinforce and protect social relationships through the development of an empathic preoccupation for the well-being of others, especially loved ones (Hoffman, 1982, 1998; Baumeister et al., 1994; Tangney and Dearing, 2002).

Recent empirical studies have tested the predictive power of this model, showing that it is possible to separately induce AG and DG by using facial expressions combined with the internal dialog typically connected with each guilt feeling (Basile and Mancini, 2011). Moreover, neuroscientific results revealed that different neuronal networks are involved in each kind of guilt, with the insula selectively responding to DG stimuli (Basile et al., 2010). It is known that the insula is activated when self-reproach and disgust are experienced (e.g., Rozin et al., 2000), thus the selective activation of the insula could reflect the major involvement of self-reproach and self-loathing characterizing DG more than AG (Rozin et al., 2000; Basile et al., 2010). Therefore, another difference concerning the two kinds of guilt might rely on the extent to which the two emotions affect self-worth. However, only a few studies have empirically tested whether and how AG and DG independently affect decision making (D’Olimpio and Mancini, 2014; Mancini and Gangemi, 2015) and none have compared a contingent enhancement of self-worth. Thus, the current study aims at disclosing how healthy participants, induced with either AG or DG, perform on a decision-making task. If DG – but not AG – decreases participants’ self-worth, only the former should prevent participants from performing any behavior that could threaten the “Do not play God” principle. To disclose the difference between AG and DG with respect to self-worth, we contrasted the induction of the two guilt feelings with that of pride in a third group of participants. Trivially, DG and pride are antithetic with respect to self-worth, which is diminished by the former and inflated by the latter.

In the present study we used a modified version of the Ultimatum Game (UG). In the classical one-shot version of the UG (Güth et al., 1982) two players must divide a given amount of money (e.g., €10). One player proposes how to split the money by making an offer. If the responder accepts, the money is divided according to the original offer; if he/she rejects, both players receive nothing. According to standard economic models, in order to maximize his/her own payoff the responder should accept any offer. Indeed, although inequitable, any offer is better than nothing. However, in accordance with the theories of reciprocal altruism (Trivers, 1971) and inequity aversion (Fehr and Schmidt, 1999), participants systematically reject unfair offers below 20–30% of the total pot (Nowak, 2000; Camerer, 2003), preferring to gain nothing rather than accepting an unequal distribution of resources (Fehr and Camerer, 2007). This type of behavior is also known as *altruistic punishment*, because it is aimed at preventing selfish free riders from adopting a similar behavior with a member of the same social group (Fehr and Gächter, 2002).

To measure how altruistic tendencies and aversion to break deontological norms differently affect decision making we expanded on previous studies in behavioral economics, which used economic games as experimental settings. In particular, the UG has been repeatedly used to examine fairness perception, prosocial motives and responses to unfairness (Camerer and Fehr, 2004; Sanfey, 2007), also under the effect of several negative emotions such as anger (Andrade and Ariely, 2009), sadness (Harlé and Sanfey, 2007), and disgust (Moretti and di Pellegrino, 2010). Additionally, the link between self-conscious emotions such as guilt (Ketelaar, 2004, 2005; de Hooge et al., 2007; Nelissen et al., 2007) or shame (de Hooge et al., 2008) and prosocial-behavior was extensively investigated by means of repeated games (e.g., the public good game, see Ketelaar and Au, 2003). While on the one hand previous literature provides a useful basis for comparison to our results, on the other hand it has failed to consider guilt not only in interpersonal terms but also in deontological terms. For this reason in the present study we used the *third-party* version of the UG. In this version participants must decide to accept or reject the offers on behalf of a third party (Civai et al., 2010; Corradi-Dell’Acqua et al., 2013). Recently it has been demonstrated that although physiological indexes of arousal were lower when participants played on behalf of a third party, they kept rejecting unfair offers (Civai et al., 2010). Hence, this version of the UG represents a valid method to investigate participants’ equity motives not just on an interpersonal axis, which contrasts selfishness and altruism but on an axis which allows for investigating the human tendency to stick to deontological norms beyond one’s own payoff.

On the basis of the described model (Mancini, 2008) we made two main predictions. The model implies a reduction of self-worth following DG but not AG induction. Therefore our first prediction was that deontological individuals (but not altruistic ones) will present a higher acceptance rate of unfair offers as compared to proud individuals, pursuing the aim of not interfering with the natural course of events and following the “Do not play God” principle. Secondly, if equity motives drive altruistic participants’ behavior, we should observe a higher amount of altruistic punishment in this group as compared to deontological participants (i.e., a lower acceptance rate of unfair offers).

## Materials and Methods

### Participants

A sample of 75 healthy participants volunteered for the study (*F* = 62; Mean age = 31.75 (±7); age range = 26–49). Participants were postgraduate students in their first year of the school for cognitive therapy (SPC) program in Rome. They were naive to the purposes of the study. Each participant was randomly assigned to one of three experimental groups: (i) DG induction (*n* = 25); (ii) AG induction (*n* = 25) and (iii) pride induction (*n* = 25). Informed consent was obtained from each participant before the experimental phase. Each participant was provided with an envelope containing a demographic questionnaire investigating age, sex, marital status, education level, and two questions assessing the presence of a neurological disorder, a psychiatric diagnosis and present or past use of psychiatric drugs. None of the participants reported neurological or psychiatric disorders or the use of psychiatric drugs. The experimental protocol was approved by the local scientific committee of SPC and was performed in accordance with the ethical standards of the 1964 Declaration of Helsinki.

### Procedure

Participants were tested in groups, during three separate sessions (i.e., one for each group). The experimenter informed participants that they were going to perform a paper and pencil questionnaire about emotions in everyday life. The paper and pencil procedure has been used in previous studies differentiating self-conscious emotions such as shame, guilt (Smith et al., 2002), and embarrassment (Tangney et al., 1996). Moreover group administration has been proven to be effective in the induction of guilt in general (Smith et al., 2002) and in the independent induction of AG and DG (Basile and Mancini, 2011). The experimenter instructed participants to remain silent for the entire duration of the experiment and to avoid any interaction. Participants filled in 9 Visual Analog Scales (VAS) to assess baseline mood. Once all participants completed the VAS, the emotion induction phase began. Right after the induction, participants were asked to again assess their mood on the same type of VAS. Finally, participants were provided with the *Third-Party Ultimatum Game Questionnaire*, which was created *ad hoc*. At the end of the experimental session participants were fully debriefed on the purposes of the study.

### Emotion Induction

Altruistic guilt and deontological guilt inductions consisted of reading two stories evoking the corresponding emotion. The stories were taken from a preliminary stage of research in a previous study (D’Olimpio and Mancini, 2014). Specifically, they were selected by independent judges from a series of personal recalls provided by 120 undergraduate students. Participants were instructed to begin reading at the same time and the induction took place simultaneously for all of them. The group procedure was also successfully adopted in a previous study, in which DG and AG were independently induced (Basile and Mancini, 2011). The order of the stories was randomized across participants. To increase the power of the induction at the end of the stories participants were further asked to recall a life event in which they had felt the same way, remembering their emotional experience. For pride induction participants were asked to recall a life event in which they felt proud of themselves remembering their emotional experience and writing it down.

### Emotion Induction Check

To assess the effectiveness of induction procedures, before and after the induction participants responded to several questions investigating the intensity of their different emotions. To conceal our interest in specific affective states, and in order to assess putative emotional halo effects, we included nine different emotions: shame, sadness, fear, disgust, anger, compassion, AG, DG, and happiness. The nine VAS scales were built following the appraisal theory of emotions, according to which specific cognitions are important antecedents of specific emotions and thereby of specific action tendencies (Smith and Ellsworth, 1985; Oatley and Johnson-Laird, 1987, 1992; Frijda et al., 1989; Scherer, 1999, 2001). Therefore, each affective state was identified by the name (e.g., DG), the corresponding mental state represented by an internal dialog sentence (e.g., “Oh God! What have I done! How dare I!?”), and the corresponding action tendency (e.g., desire to repent, to confess, to ask for forgiveness). Participants could rate the intensity of each emotion by making a mark with their pen on a VAS scale measuring 11 cm, ranging from 0 (not at all) to 100 (very much).

### Third Party Ultimatum Game Questionnaire

Right after the emotion manipulation check, participants were provided with six hypothetical dilemmas. Each dilemma described a typical UG scenario in which two people must divide a sum of money. One of the two characters was described as the proposer and the other as the responder. Participants were instructed to play on behalf of the responder. Here is an example of the UG scenarios (translated from Italian):

Anna and Marco are brother and sister. They inherited a sum of money from their grandparents. The only condition for inheritance is that Anna must propose how to split the money. You have been called upon to decide on behalf of Marco. If you accept the offer proposed by Anna, the money will be divided according to the offer. If you refuse neither Anna nor Marco will gain anything. Anna made the following offer: 60% Anna, 40% Marco.

Offers were expressed as percentages of the total pot (i.e., 90:10, 80:20, 70:30, 60:40, 50:50, and 40:60). Indeed, it has been shown across various parameters and participant pools that rejection behavior in the UG is independent from the actual amount of money offered, and rather more affected by its relative value (Camerer, 2003). Each dilemma was associated with two different offers. Therefore, each type of offer was repeated twice for a total of 12 responses. Participants could respond by writing an A (acceptance) or an R (rejection) near each offer. Moreover, they could rate the fairness of the offer on a VAS scale ranging from 0 (*not at all fair*) to 100 (*very fair*). The order of the dilemmas and that of the offers was randomized across participants.

### Data Analysis

A research assistant, who was blinded to the purpose of the study, coded all paper data. Since our data were not normally distributed across groups, as assessed by Shapiro–Wilk’s test (all *P*s < 0.05), we employed non-parametric statistics.

#### Emotion Induction

For each subject VAS scores were measured in centimeters. To assess emotion induction effectiveness, pre and post induction VAS scores for each emotion were compared using the Wilcoxon signed-rank test in each of the three groups.

Furthermore, data coding revealed that eight participants originally coded as deontological reported a higher increase in AG intensity as compared to DG (+8.50, +9.70, +3.40, +2.90, +2, +5.50, +8, +2, respectively), even if they received the DG induction. Analogously, one participant originally coded as altruistic and one participant originally coded as proud, reported higher increase in DG scores as compared to the increase in AG and pride (+1, +8, respectively). Therefore, groups were re-coded accordingly and subsequent data analysis was performed on the following groups: Deontological group (*n* = 19), Altruistic group (*n* = 32) and Proud group (*n* = 24).

In order to assess the amount of change produced by the induction with respect to the baseline, an index was obtained by subtracting the pre-induction scores from the post-induction scores. The obtained indexes for each emotion were then compared between the three new groups, by means of the Kruskal–Wallis *H* test. Where appropriate *post hoc* pairwise comparisons were performed by means of the Mann–Whitney *U* test with a Bonferroni correction for multiple comparisons. Adjusted *p*-values are reported for significant comparisons.

#### Median Acceptance and Fairness Ratings

Offers were grouped in Unfair (10:90 and 20:80), Moderately Unfair (30:70 and 40:60), and Fair (50:50 and 60:40). A Kruskal–Wallis *H* test was run to test for differences between the three groups in the distribution of acceptance of each type of offer (i.e., 10, 20, 30, 40, 50, and 60%). Where appropriate *post hoc* pairwise comparisons were performed by means of the Mann–Whitney *U* test with a Bonferroni correction for multiple comparisons. Adjusted *p*-values are reported for significant comparisons. The same type of analysis was performed on fairness ratings.

Additionally, six cumulative odds ordinal logistic regressions with proportional odds were run to determine the effect of the post induction modulation of each of the nine emotions (shame, sadness, fear, disgust, anger, compassion, AG, DG, happiness) on each type of offer (i.e., 10, 20, 30, 40, 50, and 60%). Significant results were adjusted by means of the Bonferroni correction. Where appropriate adjusted *p*-values are presented.

## Results

### Induction Check

As shown in **Table 1**, the Wilcoxon signed-rank test, run on the original groups, those with 25 participants exposed to the three conditions, determined that the median post-induction scores related to DG and AG were higher as compared to the median pre-induction-scores in DG and AG groups, respectively. Furthermore, the proud group showed a significant increase in happiness scores (all *P*s < 0.0001). Proud participants’ recalls are shown in **Table 2**.

**Table 1:** Pre and Post induction scores for each group, Wilcoxon *Z* and significance levels (^∗^*p* < 0.05; ^∗∗^*p* < 0.01; ^∗∗∗^*p* < 0.001).

Deontologic Guilt (DG) group	Med. Pre	Med. Post	*Z*(24)	*p*
Shame	0	2.5	4.166	0.00031^∗∗∗^
Sadness	0	2.2	3.485	0.000491^∗∗∗^
Fear	0	5.2	3.845	0.000120^∗∗∗^
Disgust	0	0.4	3.407	0.000655^∗∗^
Anger	0	0	1.765	0.077
Compassion	0	5.5	4.057	0.00005^∗∗∗^
Altruistic Guilt (AG)	0	5.1	3.847	0.00012^∗∗∗^
**DG**	**0**	**5.20**	3.919	**0.00009^∗∗∗^**
Happiness	4	0	3.562	0.00037^∗∗∗^

**AG group**	**Med. Pre**	**Med. Post**	***Z*(24)**	***p***

Shame	0	0.9	0.958	0.338
Sadness	0.3	2.2	2.165	0.030^∗^
Fear	1	0.8	2.532	0.011^∗^
Disgust	0	0.5	3.283	0.001^∗∗∗^
Anger	1	4.8	4.130	0.000036^∗∗∗^
Compassion	1.5	8	3.968	0.000072^∗∗∗^
**AG**	**0.3**	**6**	**3.985**	**0.000067^∗∗∗^**
DG	0.2	0.30	1.111	0.266
Happiness	7.2	0.1	4.210	0.000025^∗∗∗^

**Proud group**	**Med. Pre**	**Med. Post**	***Z*(24)**	***p***

Shame	0	0	1.65	0.10
Sadness	1	0	2.187	0.028^∗^
Fear	0.4	0	1.111	0.266
Disgust	0	0	0.070	0.944
Anger	0	0	0.90	0.366
Compassion	0	0	1.725	0.084
AG	0	0	0.800	0.423
DG	0	0	1.070	0.284
**Happiness**	**6**	**8.4**	**4.049**	**<0.00005^∗∗∗^**

*Target emotions in each group and their significance values are displayed in bold*.

**Table 2:** The Table reports pride episodes recalled by the 25 participants in the original proud group.

	Pride Stories
1	*I was proud of myself when during my third year at University I have done everything I could not to lose a scholarship. I passed many exams in a few days. This allowed me to pay for my studies and not to weigh on my family.*
2	*I was proud of myself when I gave a lecture about communication techniques. Participants showed interest, asking many questions and requested an additional class.*
3	*Two weeks ago I quit a job that was recently causing me physical and psychological suffering. A disgusting job, against my personal values. I had the strength to say “Enough!” I saved myself.*
4	*I was proud of myself when I was offered a job in the place where I had done an internship. I was very proud because I was chosen for my abilities and not for some kind of connection.*
5	*Last Thursday I went to the retirement home where I work as a volunteer. I prepared a CD with old songs to entertain the elderlies. They were all very happy: they sang and danced. I felt happy and satisfied because I was cheering up their day and I was gaining consideration and success from that. Doing something for the elderlies made me happy!*
6	*To be able to follow a diet and lose the weight that I had planned: pride and a sense of power and control.*
7	*Some years ago I took a carrier path that seemed profitable and in line with my studies and interests. I completed a work that made me proud. With time the opportunities connected with this job have downsized but what remains is a sense of “know how” that still makes me proud.*
8	*I offered a patient a free therapy session because she had financial difficulties.*
9	*I felt proud last time I made the right diagnosis.*
10	*I was proud listening the recording of my radio program. Although I am not a professional the program has many followers.*
11	*The first time in Rome with my wife. Driving through the city at night and realizing that I made a dream come true.*
12	*During therapy I was able to cooperate with a patient who was very resistant. She shared her realty with me. I was satisfied for having done a good job.*
13	*My graduation day: a feeling of pride and happiness that I shared with friends and family.*
14	*During a meeting at work I gave a speech that was appreciated both by my boss and by the rest of the team.*
15	*I received praise and gratitude for a voluntary work.*
16	*Some weeks ago I arranged a meeting between two of my friends who barely talked to each other. When the two met they hugged themselves crying. I felt proud of myself, because thanks to me my two friends talked, and solved their problem.*
17	*After several disappointing years, I gave my first lecture in a research center obtaining a discrete amount of success. The feeling of compensation for all my efforts and the signs of appreciation from the audience gave me real joy and made me proud.*
18	*When I signed a job contract. I was very happy and proud of myself.*
19	*Today the husband of a patient thanked me because I treated his wife not only professionally but also with kindness. The patient had a threat of miscarriage after three miscarriages. I felt proud not only for his acknowledgment but especially because I was able to combine, in treating that patient, both technique and kindness.*
20	*A few months ago the head of the department where I work gave me the responsibility to develop a research proposal that, if approved, would have brought funding to the whole department. After I sent him the project proposal he came looking for me to congratulate for having done a good job. I felt very proud of myself, and I felt my commitment to be acknowledged.*
21	*When I graduated I felt proud and satisfied. Although I made many sacrifices, I achieved one of my goals and I felt happy and impatient to start working*
22	*When I graduated. I felt happiness, satisfaction, a sense of achievement and self-efficacy.*
23	*I was proud for having done a good job with a child in therapy.*
24	*I felt proud of myself when during a therapy session the patient told me that he was feeling better and that the work I had done was helpful.*
25	*The first time I took an airplane it was for an 11 h flight. Although I was a little scared, I felt very proud.*

The Kruskal–Wallis *H* test, run on the three newly coded groups, revealed that the medians of the increase index scores were statistically significantly different between groups (**Table 3**). Distributions of the increase indexes were similar for all groups, as assessed by visual inspection of a boxplot.

**Table 3:** Kruskal–Wallis *H* test results for each emotion, with χ^2^ values and asymptotic significance (two-sided test).

Emotion	χ^2^(2)	Asympt. *p*
Shame	14.080	0.001
Sadness	18.617	0.000
Fear	17.091	0.000
Disgust	19.254	0.000
Anger	18.745	0.000
Compassion	34.122	0.000
AG	30.774	0.000
DG	31.964	0.000
Happiness	43.344	0.000

Mann–Whitney *U* test for pairwise comparisons revealed that DG showed a higher increase in the deontological group (Median = 8.5) with respect to the altruistic group (Median = 0, *Adj.p* = 0.0003) and to the proud group (Median = 0, *Adj.p* = 0.0003). No significant differences were found between the altruistic group and the proud group in the increase of DG (*p* = 0.17, NS). Shame was increased to a higher extent in the deontological group (Median = 0.9) as compared to the proud group (Median = 0, *Adj.p* = 0.0003). No significant differences were found between the altruistic group and the proud group in the increase of shame (*p* = 0.124, NS). Fear increased more in the deontological group (Median = 1.8) than in the proud group (Median = 0, *Adj.p* = 0.0003). Additionally fear increase was higher in the altruistic group (Median = 1.1) as compared to the proud group (*Adj.p* = 0.0003).

Altruistic guilt showed a higher increase in the altruistic group (Median = 4.65) with respect to both the deontological group (Median = 1.2, *Adj.p* = 0.021) and the proud group (Median = 0, *Adj.p* = 0.0003). Additionally, AG was higher in the deontological group with respect to the proud group (*Adj.p* = 0.018). Compassion showed a higher increase in the altruistic group (Median = 5.7) with respect to both the deontological group (Median = 1.8, *Adj.p* = 0.042) and the proud group (Median = 0, *Adj.p* = 0.0003). Additionally, the deontological group showed a higher increase in compassion scores than the proud group (*Adj.p* = 0.0003). Furthermore, the increase in anger was higher in the altruistic group (Median = 2.150) as compared to both the deontological group (Median = 0, *Adj.p* = 0.0003) and the proud group (Median = 0, *Adj.p* = 0.021). In addition the median anger increase was not different between the deontological and the proud group (*Adj.p* = 0.153, NS).

Sadness showed a lower increase in the proud group (Median = –0.50) with respect to both the deontological group (Median = 1.9, *Adj.p* = 0.0003) and the altruistic group (Median = 0.5, *Adj.p* = 0.0003). Disgust showed a lower increase in the proud group (Median = 0) with respect to both the deontological group (Median = 5, *Adj.p* = 0.0003) and the altruistic group (Median = 0.5, *Adj.p* = 0.009). The median increase in shame, disgust, fear and sadness were not significantly different between the deontological group and the altruistic group (all *P*s > 0.103).

**FIGURE 1:**
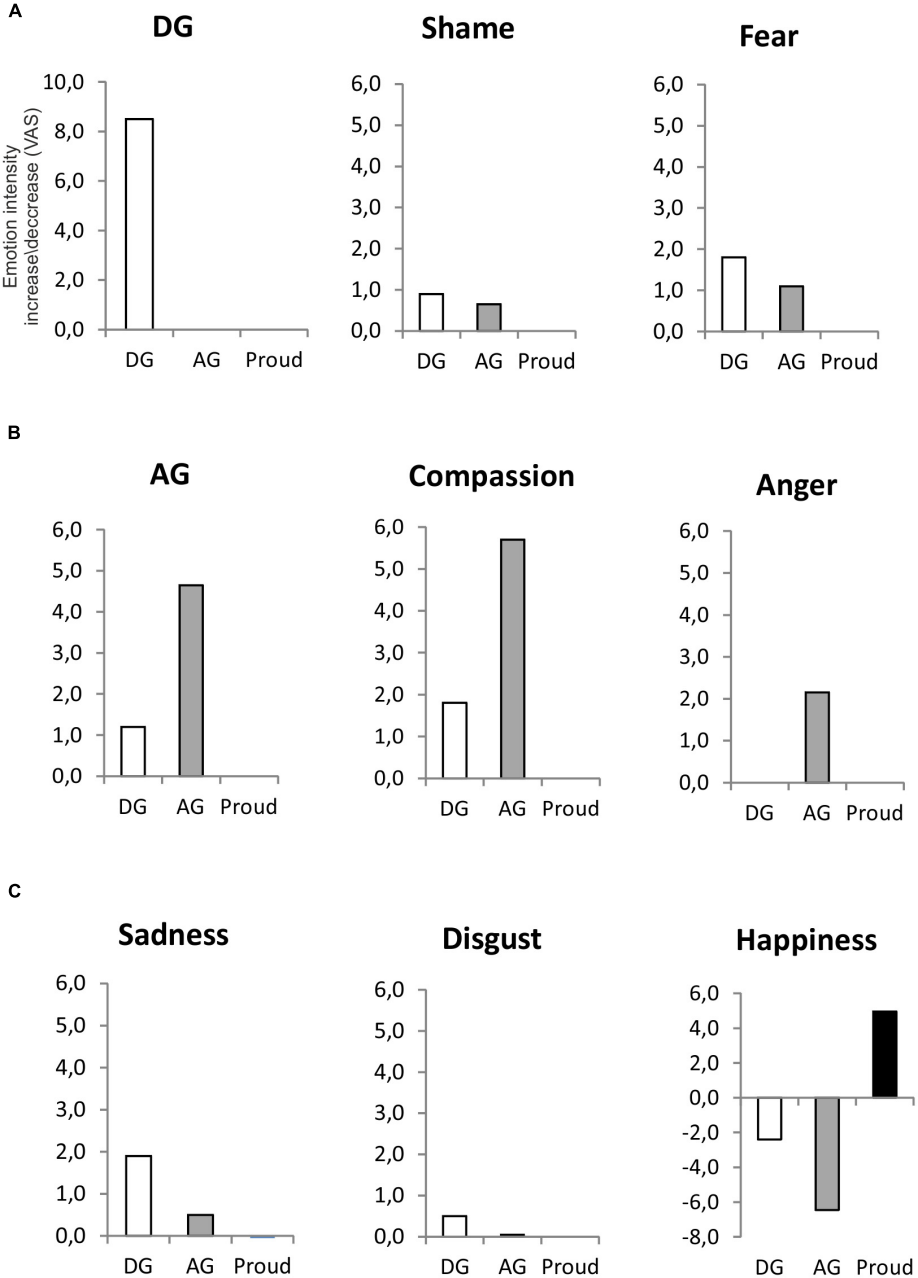
**The figure shows the median increase of each emotion after the induction for the deontological group (white bars), altruistic group (gray bars) and proud group (black bars). (A)** The emotions presenting a higher median increase in the deontological group than in the proud group [namely deontologic guilt (DG), shame and fear]. **(B)** The emotions that presented a higher median increase in the altruistic group with respect to both the deontological and the proud group [namely altruistic guilt (AG), compassion and anger]. **(C)** The emotions presenting similar increase in the AG and DG group and happiness scores (which showed a higher decrease in the AG group with respect to the other two groups.

Finally happiness increased to a higher extent in the proud group (Median = 4.95) both with respect to the deontological group (Median = –24, *Adj.p* = 0.0003) and to the altruistic group (Median = –6.45, *Adj.p* = 0.0003). Additionally, median happiness increase index was lower in the altruistic group than in the deontological group (*Adj.p* = 0.036). Differences in median values for each emotion between groups are represented in **Figure 1**.

### Median Acceptances between Groups

**FIGURE 2:**
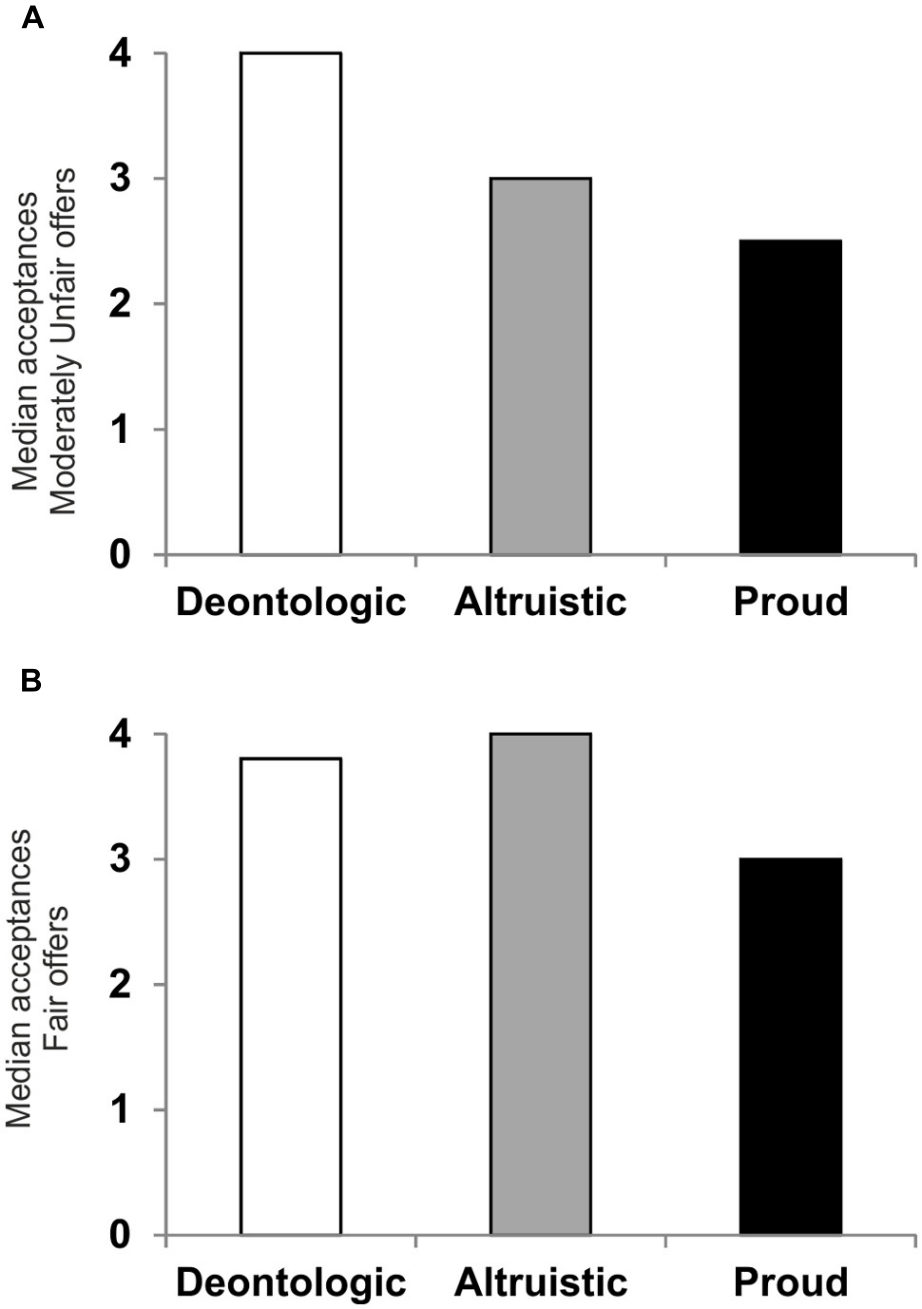
**(A)** Median acceptance of Moderately Unfair offers presented by the deontological group (white bar), the altruistic group (gray bar), and the pride group (black bar). **(B)** Median acceptance of Fair offers presented by the deontological group (white bar), the altruistic group (gray bar), and the proud group (black bar).

**Figure 2** illustrates median acceptances as a function of the UG offers and emotion condition.

The distributions of acceptance had a similar shape for all groups, as assessed by visual inspection of the boxplots. The Kruskal–Wallis *H* test showed that for Moderately Unfair offers, median acceptance was statistically significantly different between groups, [χ^2^(2) = 7.567, *p* = 0.023]. The Mann–Whitney *U* test for pairwise comparisons revealed that median acceptance for the deontological group was higher than that of the proud group (*Adj.p* = 0.027; **Figure 2A**). Neither the comparison between the altruistic group and the proud group, nor that between the deontological group and the altruistic group were significantly different (*p* = 0.130, and *p* = 0.096, respectively). Moreover, for Fair offers, median acceptance values were significantly different between groups, [χ^2^(2) = 6.385, *p* = 0.041]. The Mann–Whitney *U* test for pairwise comparisons revealed that median acceptance of the altruistic group was higher than that of the proud group (*Adj.p* = 0.042; **Figure 2B**). Neither the comparison between the deontological group and the proud group, nor that between the deontological group and the altruistic group were significantly different (*p* = 0.144 and *p* = 0.337, respectively). For Unfair offers median acceptance was not significantly different between groups (*p* > 0.922).

### Fairness Ratings

The Kruskal–Wallis *H* test on fairness ratings scores showed no significant differences between groups for all the three types of offers (all *P*s > 0.285).

### Ordinal Regression

**FIGURE 3:**
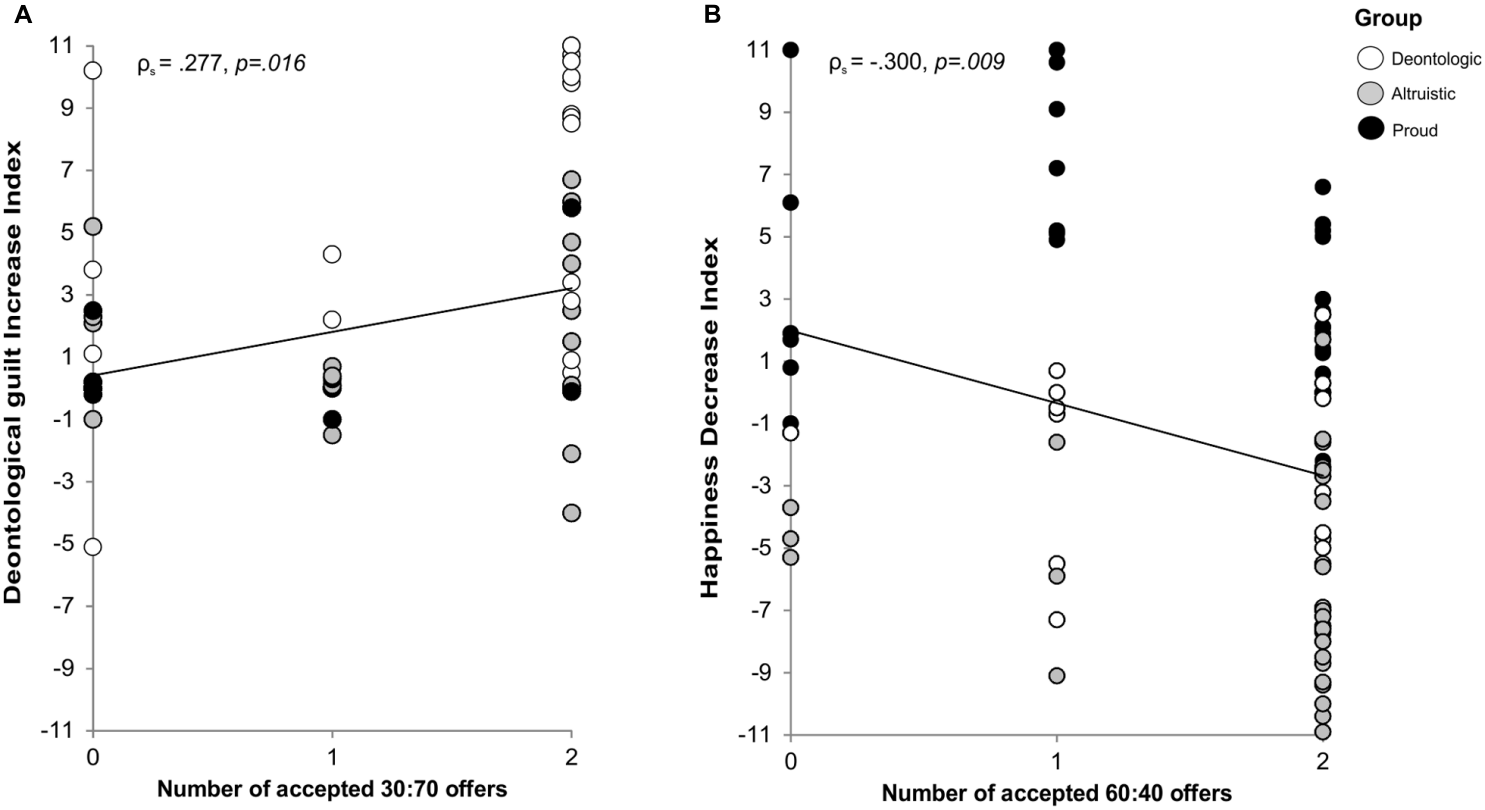
**(A)** The number of times participants accepted 30:70 offers (i.e., 0;1;2, *X*-axis) as a function of the increase in deontological guilt (*Y*-axis). Each participant is identified as deontological (white circles), altruistic (gray circles), or proud (black circles). **(B)** The number of times participants accepted 60:40 offers (i.e., 0;1;2, *X*-axis) as a function of the decrease in happiness (*Y*-axis). Each participant is identified as deontological (white circles), altruistic (gray circles), or proud (black circles). Spearman Rho correlation coefficients and their relative *p*-values are reported above each panel.

The only ordinal regression model found to be significant was for 30% offers. The assumption of proportional odds was met, as assessed by a full likelihood ratio test comparing the residual of the fitted location model to a model with varying location parameters, [χ^2^(9) = 13.266, *p* = 0.151]. The deviance goodness-of-fit test indicated that the model was a good fit for the observed data, [χ^2^(139) = 135.165, *p* = 0.576], but most cells were sparse with zero frequencies in 66.7% of cells. However, the final model statistically significantly predicted the dependent variable over and above the intercept-only model, [χ^2^(9) = 135.165, *p* = 0.011]. A higher increase in DG was associated with an increase in the odds of accepting 30% offers, with an odds ratio of 1.466 (95% CI [1.166 to 1.843]), [Wald χ*^2^*(1) = 10.717, *Adj.p* = 0.009; **Figure 3A**]. The increase indexes of other emotions were not significantly associated with participants’ responses to 30% offers (all *P*s > 0.13).

For 60% offers the deviance goodness-of-fit test indicated that the model was a good fit for the observed data, [χ^2^(139) = 115.055, *p* = 0.932], but most cells were sparse with zero frequencies in 66.7% of cells. However, the ability of the model to predict the dependent variable over and above the intercept-only model approached significance, [χ^2^(9) = 16.395, *p* = 0.059]. A higher decrease in happiness levels was associated with an increase in the odds of accepting 60% offers, with an odds ratio of 0.845 (95% CI [0.750 to 0.951], [Wald χ^2^(1) = 7.747, *Adj.p* = 0.045]. The assumption of proportional odds was met, as assessed by a full likelihood ratio test comparing the residual of the fitted location model to a model with varying location parameters, [χ^2^(9) = 14.736, *p* = 0.098; **Figure 3B**]. The increase indexes of other emotions were not significantly associated with participants’ responses to 60% offers (*all P*s > 0.14).

## Discussion

Our results showed that the induction was effective in enhancing baseline levels of the targeted emotion in each group. DG scores were higher in the deontological group as compared to the altruistic and the proud groups. However, eight participants originally coded as deontological presented a higher enhancement of AG as compared to DG and one participant originally coded as altruistic presented a higher enhancement of DG. It might be that the scenarios described in some of the stories used for the DG induction also elicited in some participants altruistic and empathic feelings (as accounted for by post-induction compassion scores, which were strongly enhanced in the original DG group). It is worth noting that previous experiments combined the use of internal dialog sentences with the exposure to emotional facial expressions (Basile and Mancini, 2011). This latter method might be more effective to separately induce the two types of guilt.

Data analysis performed on the increase indexes of the newly coded groups revealed that the enhancement in AG was accompanied by an increase in anger and compassion scores, which were greater in the AG group with respect to the other two groups. Additionally, the AG group presented a significantly higher decrease in happiness scores as compared to the other two groups. Similar emotional “halo effects” were also found in previous studies (see Basile and Mancini, 2011 relative to compassion and sadness, and D’Olimpio and Mancini, 2014 relative to anger). These effects were consistent with the goal of AG, which is to promote pro-social and reparative behaviors toward the victim and feeling sorry for her/him (Mancini, 2008; Basile and Mancini, 2011).

The main purpose of our research was to determine whether and how different guilt feelings, namely the deontological type and the altruistic type, differently affected decision making, as compared to pride in a third party version of the UG. In accordance with our first prediction, results showed that participants induced with DG accepted Moderately Unfair offers to a higher extent as compared to proud participants. This result can be explained by the opposite effect that DG and pride exert on self-worth. Indeed, recent accounts posited that factors others than immediate emotional reaction, such as the judgments directed toward the self, influence decision making in the UG (Dunn et al., 2010). Specifically, high trait positivity (i.e., the tendency to experience positive emotions rather than negative) was related to a higher rejection rate of unfair offers, while high trait negativity was related to the acceptance of such offers. Interestingly, the relationship between positive and negative trait levels and the levels of rejection of unfair offers was not mediated by contextual variations in participants’ mood. Therefore, the authors hypothesized that they were rather mediated by self-worth, with those of a positive disposition believing to be “worth more than that” and those of a negative disposition resigning themselves to “take the crumbs from under the table” (Dunn et al., 2010). Consistently, it was shown that high levels of disgust toward the self-strongly modulated moral judgments of others’ behavior. In particular, individuals with high traits of self-loathing judged strict moral transgressions (e.g., murder) as less disgusting and punishment deserving, as compared to participants with low self-loathing traits (Olatunji et al., 2012). Recent evidence has shown that AG and DG activate different brain networks. Specifically, while AG was associated with activity in medial prefrontal areas, consistently associated with theory of mind, empathy, and compassion (Blair, 1995; Shallice, 2001; Moll et al., 2006), DG activated the anterior cingulate cortex and insula, previously associated with self-reproach and disgust (Rozin et al., 2000).

An alternative explanation could be that since the induction of DG produced an increase in several other negative emotions, the increase in one of these or the general negativity of the affect experienced by deontological participants could have been predictive of their behavior in the UG. However, this interpretation seems to be disconfirmed by the results of the ordinal regression model, which showed only the increase of DG to be predictive of 30:70 offers median acceptance. In addition, fairness ratings were not different across groups, indicating that the higher acceptance rate of Moderately Unfair offers exhibited by deontological participants did not depend on a decreased ability to judge others’ behavior. According to the principle of “Do not play God” no one has the right to make a decision that does not respect the limits of his/her social rank or function (Sunstein, 2005). Therefore the adoption of this principle should induce a humble attitude, which results in the limitation of decisional autonomy (Mancini, 2008). Previous accounts have shown that DG and AG differently affect moral decision-making (Mancini and Gangemi, 2015). Specifically, participants induced with DG preferred inaction when faced with the switch version of the trolley dilemma, while those induced with AG preferred action (Mancini and Gangemi, 2015). Crucially, deontological participants justified their omission bias with the goal of not interfering with the natural order, or in other words, with the “Do not play God” principle (Gangemi and Mancini, 2013). Moreover, the presence of an authority limited participants’ decision-making autonomy activating the “Do not play God” principle and led them to prefer inaction (Gangemi and Mancini, 2013).

According to our second prediction, the induction of altruistic motives should have brought about a lower acceptance rate of unfair offers in AG participants as compared to DG participants. This would have also been consistent with the high scores of anger and the lower scores of happiness presented in the AG group with respect to the other two groups. Indeed, both angry and sad participants were significantly more likely to reject unfair offers in the classical version of the UG (Harlé and Sanfey, 2007; Andrade and Ariely, 2009). Contrastingly, altruistic participants’ median acceptance of Moderately Unfair offers was not significantly lower than that of deontological participants, nor was it higher than proud participants. One possible interpretation of this null result is that participants in the AG condition were somewhat divided between equity motives and the use of a utilitarian strategy, reflecting the aim of minimizing losses. Indeed, altruistic participants adopted a utilitarian strategy when faced with the switch version of the trolley moral dilemma (Mancini and Gangemi, 2015), choosing to “cause” the death of three people in order to save five others. Crucially, participants preferring the action tended to justify it by referring to the altruistic principle that prescribes minimizing other’s suffering (for example: “it’s better that three people die instead of five”). However, when faced with 60:40 offers, which are unbalanced in favor of the receiver, altruistic participants presented a higher median acceptance with respect to proud participants. The results of the ordinal regression suggest that these types of acceptances are compensatory, since 60:40 acceptances tended to be predicted by a decrease in happiness levels, lower in those who accepted these offers more frequently.

It is noteworthy that participants did not benefit economically from accepting the offers, since they were playing on behalf of a third party. According to social preference theory, people prefer to behave pro-socially because they derive higher hedonic value from mutual cooperation and altruism (Thibaut and Kelley, 1959; Fehr, 2008). Thus, it is widely held that the brain uses a common-reward metric for the processing of both individual and social rewards (Sanfey, 2007). Specifically, an increased activation of the ventral tegmental and striatal areas was found both when receiving money and in non-costly charitable donations (Moll et al., 2006; Harbaugh et al., 2007).

## Conclusion

In sum, while DG leads to a higher acceptance rate of Moderately Unfair offers as compared to pride, AG does not show the same effect. We hypothesize that this occurs because proud participants feel entitled enough to take action in order to restore equity, while deontological participants are prone to take a humble attitude, which does not allow them to decide on behalf of others. AG instead may not entail a change in perceived hierarchy and possible utilitarian motives are not intense enough to produce any behavioral difference with proud participants in the acceptance of Moderately Unfair offers. However, in the present study self-worth was not directly estimated. This is a limitation of our results and future studies should directly assess the effect of AG and DG on self-worth and perceived moral hierarchy.

The distinction between AG and DG is important in light of the role played by guilt in obsessive compulsive disorder (OCD). Recent findings have shown that DG and not AG is the mental state underlying checking and cleaning compulsions (D’Olimpio and Mancini, 2014). Additionally, fMRI results have revealed an abnormal processing of DG (but not AG) in OCD patients (Basile et al., 2013). Finally, when faced with moral dilemmas, OCD patients – but not anxious patients – preferred omission, presumably in order to respect the of “Do not play God” principle (Mancini and Gangemi, 2015).

## Author Contributions

Conceived and designed experiment: AM, FM. Data collection: AM, Alessia di Febo. Data Analysis: AM, FM. Wrote the paper: AM, FM.

## Conflict of Interest Statement

The authors declare that the research was conducted in the absence of any commercial or financial relationships that could be construed as a potential conflict of interest.
